# Disadvantaged groups have greater spatial access to pharmacies in New York state

**DOI:** 10.1186/s12913-024-10901-8

**Published:** 2024-04-15

**Authors:** Abhinav Suri, James Quinn, Raymond R. Balise, Daniel J. Feaster, Nabila El-Bassel, Andrew G. Rundle

**Affiliations:** 1 Columbia University Mailman School of Public Health, New York, NY United States of America; 2 David Geffen School of Medicine at UCLA, Los Angeles, CA United States of America; 3 Department of Public Health Sciences, University of Miami Miller School of Medicine, Miami, FL United States of America; 4 Columbia University School for Social Work, Columbia University, New York, NY United States of America

**Keywords:** Pharmacy, Accessibility, Geospatial, Socio-economic, Disadvantaged, Census

## Abstract

**Background:**

The accessibility of pharmacies has been associated with overall health and wellbeing. Past studies have suggested that low income and racial minority communities are underserved by pharmacies. However, the literature is inconsistent in finding links between area-level income or racial and ethnic composition and access to pharmacies. Here we aim to assess area-level spatial access to pharmacies across New York State (NYS), hypothesizing that Census Tracts with higher poverty rates and higher percentages of Black and Hispanic residents would have lower spatial access.

**Methods:**

The population weighted mean shortest road network distance (PWMSD) to a pharmacy in 2018 was calculated for each Census Tract in NYS. This statistic was calculated from the shortest road network distance to a pharmacy from the centroid of each Census block within a tract, with the mean across census blocks weighted by the population of the census block. Cross-sectional analyses were conducted to assess links between Tract-level socio demographic characteristics and Tract-level PWMSD to a pharmacy.

**Results:**

Overall the mean PWMSD to a pharmacy across Census tracts in NYS was 2.07 Km (SD = 3.35, median 0.85 Km). Shorter PWMSD to a pharmacy were associated with higher Tract-level % poverty, % Black/African American (AA) residents, and % Hispanic/Latino residents and with lower Tract-level % of residents with a college degree. Compared to tracts in the lowest quartile of % Black/AA residents, tracts in the highest quartile had a 70.7% (95% CI 68.3–72.9%) shorter PWMSD to a pharmacy. Similarly, tracts in the highest quartile of % poverty had a 61.3% (95% CI 58.0-64.4%) shorter PWMSD to a pharmacy than tracts in the lowest quartile.

**Conclusion:**

The analyses show that tracts in NYS with higher racial and ethnic minority populations and higher poverty rates have higher spatial access to pharmacies.

**Supplementary Information:**

The online version contains supplementary material available at 10.1186/s12913-024-10901-8.

## Introduction

Accessibility to healthcare is a fundamental predictor of overall wellbeing in the United States [1]. Pharmacies are among the many instruments of the healthcare system individuals interact with on a frequent basis. Across the nation, pharmacies provide people consistent access to not only prescription medications, but also many other health related goods such as non-prescription/over-the-counter medications, contraception, and other medical devices [2]. Thus, pharmacies play an important role in health to the individuals in its surrounding community.

Additionally, pharmacies serve to address health beyond the level of the individual. Pharmacies serve as the centers for more direct healthcare interventions and education as well, functioning as bases for efforts to manage hypertension, diabetes, and many other chronic diseases in a cost-effective manner [3–5]. For example, during the COVID-19 pandemic, U.S. pharmacies provided a critical source for millions of clinical & home COVID-19 tests [6–10]. Conversely, pharmacies have also played a complex role in the opioid epidemic having dispensed, sometimes inappropriately, millions of doses of opioids, but also dispensing medications for opioid use disorder [11, 12]. Yet pharmacies also play a key role in addressing this epidemic through harm reduction efforts that distribute clean syringes and directly save lives through the distribution of naloxone [13]. Lastly, pharmacy-based community initiatives have also been shown to be instrumental in the development of opioid stewardship initiatives where education initiatives and medication therapy adjustments can play a role in minimizing the incidence of opioid dependence in communities [14]. It is clear that pharmacies have played a major role in public health trends over the past several years.

Despite their prevalence, pharmacies are not always accessible and it is unclear who actually benefits the most from pharmacies. Fundamental Cause Theory (FCT) argues that associations between socioeconomic status (SES) and health status continuously recapitulate themselves because higher SES encompasses an array of resources such as money, knowledge and access that promote health regardless of what mechanisms are relevant at any given time [15]. Access to neighborhood-level resources may be a mechanism through which FCT operates, in that higher income neighborhoods may attract some health promoting businesses and residents may have a higher capacity to create health promoting resources (e.g. fund parks) or block the siting of environmental disamenities (e.g. factories). Just as researchers have linked the existence of “food deserts” to areas of socioeconomic disadvantage, so too have researchers endeavored to show a similar association between “pharmacy deserts” (defined as areas with increased travel distance to pharmacies) and disadvantaged neighborhoods and neighborhoods of color [16–18]. However, the literature linking “pharmacy deserts” to specific neighborhood sociodemographic characteristics (such as higher numbers of Black or Hispanic residents) has shown inconsistent results and is less consistent than the literature on food deserts [18].

Given that there is ambiguity regarding the prevalence of pharmacy deserts beyond the city level, we aim to address this shortcoming of the prior literature. In this study, we seek to describe disparities in spatial access to pharmacies across New York State, a highly populated, and diverse state. Based on FCT, we hypothesize that higher SES Census-tracts will have shorter population-weighted road network distances to pharmacies. To address this research objective, we use a novel, population-weighted measure of road network distance to pharmacies in New York State to assess Census tract-level disparities in spatial access to pharmacies. Our method accounts for “border effects” by including pharmacies in nearby areas of bordering states and analyzes data for the whole state and for tracts inside and outside of New York City separately.

## Materials and methods

### Geographic pharmacy dataset creation

Pharmacy locations in New York State were extracted from the New York State Board of Pharmacy Dataset. Data from RX Open (https://rxopen.org), which provides a comprehensive list of pharmacies in the United States, were used to identify pharmacies in states neighboring New York State [19, 20]. Pharmacies in Canada (which shares a border with NY State) were excluded due to the fact that Canadian pharmacies likely would not be accessible to individuals given international policies limiting the utilization of prescriptions from across the border.

For each 2010 Census tract we estimated a population weighted mean distance to the nearest pharmacy using ArcGIS Pro v2.9 [21]. To do this, the shortest road network distance to a pharmacy from the geographic centroid of each Census block in NYS was calculated. Using the population counts for each Census block, the population weighted mean shortest distance was calculated across Census blocks within each tract (see Supplemental Sect. 1). This tract-level measure generated from the Census blocks within each tract, provided a population weighted measure of the spatial access to pharmacies for the population of each Tract. It is thought that this approach reflects the population experience of spatial access to pharmacies for residents of each Tract.

**Fig. 1:**
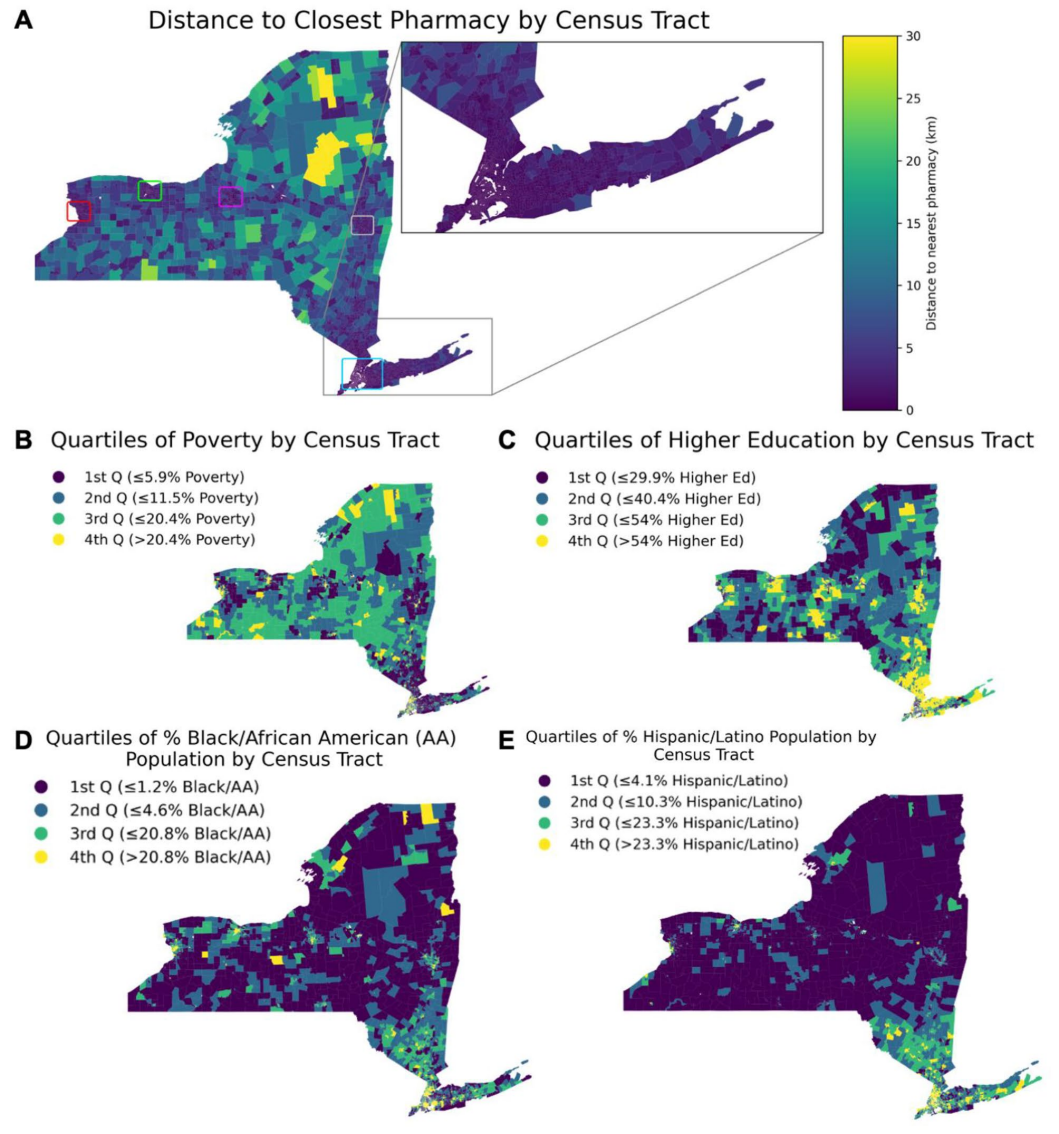
Maps of NYS by Census Tract. In each of the panels, we show the census tracts across NY State according to a 5 primary descriptors. In Panel **A**, we show the distance of census tracts to the nearest pharmacies in km. Additionally, major cities areas are outlined in rectangles (red = Buffalo, light green = Rochester, magenta = Syracuse, gray = Albany, light blue = New York City). In Panel **B**, we show the census tracts stratified by quartiles of poverty (1st quartile = least poverty, 4th quartile = highest poverty). In Panel **C, D**, and **E**, we show the quartiles of % of the population in that census tract with higher education (i.e. anything greater than or equal to an associate’s degree), Hispanic/Latino origin, or Black/African American Origin, respectively. For these panels, a 1st quartile census tract indicates the smallest % of individuals with that trait and the 4th quartile has the largest % of individuals with that trait. Shapefiles from https://catalog.data.gov/dataset/tiger-line-shapefile-2018-state-new-york-current-census-tract-state-based

The 2018 Topologically Integrated Geographic Encoding and Referencing Line Files were downloaded from data.census.gov and used to define tract and block boundaries [22]. American Community Survey (2014–2018; 5-year estimate) data were used to describe the sociodemographic characteristics of each Census tract. Sociodemographic variables considered in the analyses were: proportion of residents living in poverty (per table B17001 of ACS estimates), proportion of residents with higher educational attainment (associates, bachelor’s, master’s, professional school, or doctorate degrees), proportion of the residents who were Black or African American Alone, and the proportion of the residents who were Hispanic or Latino. The finalized dataset is available at the following link: https://drive.google.com/file/d/1YbQgv2MRCxG9QGRiNFQjIuXHa0D3JbIq/view?usp=share_link. A geographical visualization of the distribution of these variables can be found in Fig. 1.

### Exclusion criteria and statistical analysis

The dataset initially included 4,918 NYS Census tracts. Eighteen of the Census tracts were removed from the dataset as they contained no land area, only water, resulting in 4900 total census tracts. Additionally, census tracts with less than 10 people were removed due to our inability to determine an accurate population weighted distance to the nearest pharmacy. The final dataset therefore had 4844 Census tracts available for analysis (i.e. 56 tracts removed due to low population). On average, Census tracts had 4050 ± 1913 residents (± 1 standard deviation).

The goal of these analyzes is to document disparities in access to pharmacies and so extensive multivariate analyses of confounders and mediators were not conducted as primary analyses, although in the Supplemental materials we present results adjusting for population density and assessing spatial autocorrelations. The dependent variable in our analysis was the population weighted mean shortest distance (PWMSD) to pharmacies which was natural log (Ln) transformed to generate a more normal distribution for the outcome variable. Tract level data on each of the sociodemographic variables, % poverty, % higher education, % Black/AA, % Hispanic/Latino, were categorized into quartiles using the distribution of the variables across tracts in NYS. Cut points for quartiles are presented in the Legend of Fig. 1, panels B, C, D, and E. Generalized linear models (GLM) with robust standard errors (implemented in the sandwich and lmtest R v4.0.3 packages) were used to assess associations between the quartiles of tract-level variables and the Ln transformed population weighted shortest distances to a pharmacy, with separate models fit for each of the predictor variables [23–25]. Robust standard error estimation was used to account for the non-independence of observations. The first quartile of each tract level variable as used as the referent group in the GLM analyses. Beta coefficients from the GLM models were converted into % differences in the distance to aid in interpretation of analyses of the Ln transformed dependent variable.

## Results

Overall the mean PWSMD to a pharmacy across Census tracts in NYS was 2.07 ± 3.35 Km (± 1 SD; median: 0.85 Km). 2703 out of 4844 tracts (55.8% of tracts) had a shortest PWSMD to the nearest pharmacy of < 1 Km and 10,720,881 people out of 19,616,336 (54.6%) of the population lives in these tracts. The maximum PWSMD in our dataset was 52.4 Km. Distances were typically shorter in urban areas such as New York City, Albany, Syracuse, and Buffalo (ref Fig. 1, panel A, boxed areas). Among the top 10% of census tracts by population, the mean PWSMD to a pharmacy was 1.47 ± 1.73 Km (± 1 SD; median: 0.81 Km). Among the bottom 10% of census tracts by population, the mean PWSMD to a pharmacy was 2.38 ± 4.84 Km (± 1 SD; median: 0.87 Km).

**Fig. 2:**
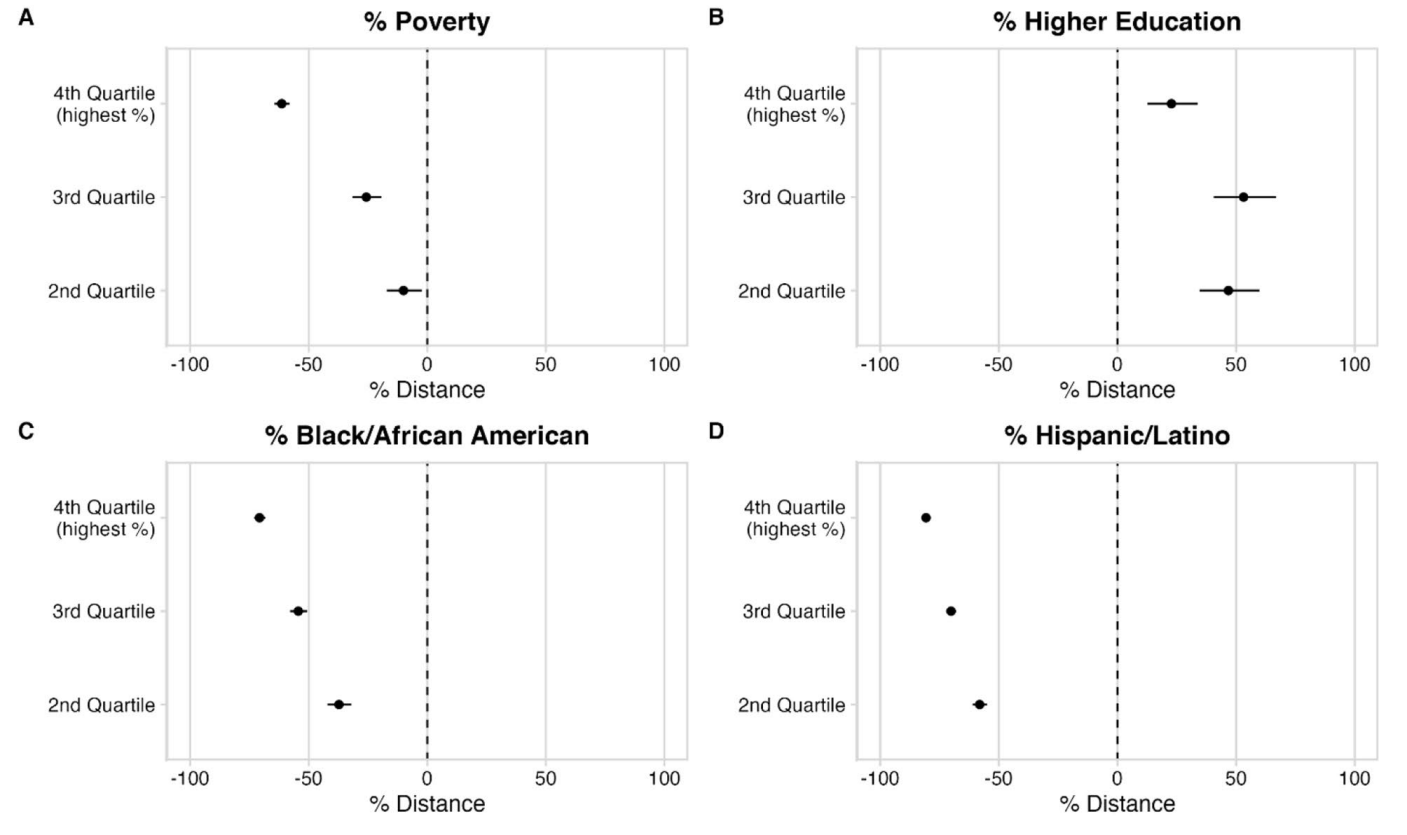
Coefficients plotted out w/95% CIs. For each of the characteristics examined (**A** = % poverty, **B** = % higher education, **C** = % Black/African American, **D** = % Hispanic/Latino) from 5-year estimate ACS data across 4844 census tracts in New York State, we plot the % differences in distance to pharmacies from our generalized linear model analysis. Since 1st quartile was treated as the reference level in our analyses, this level was excluded from this graph. Null effect is indicated with the vertical dashed line and the coefficients with associated 95% CIs are plotted (dot = coefficient value, lines = 95% CI).

There were substantial difference in PWSMD across tracts by racial and ethnic composition and by poverty rate (see Table 1; Fig. 1). The maps in Fig. 1 show that there is substantial regional clustering of populations by race and ethnicity in NYS, with high proportions of Hispanic and Black residents in the South East of the state, an area of higher urbanization, population density and retail density. As such, tracts with greater proportions of residents who are Black/AA or Hispanic have shorter PWSMD to pharmacies, suggesting greater spatial access to pharmacies. We formally display the % difference in PWSMD to pharmacy by quartile of the analyzed sociodemographic characteristics in Fig. 2 (and also report findings numerically in Table 2). For instance, tracts that were in a higher quartile of poverty (i.e. higher percentages of residents living in poverty) had shorter PWSMD to pharmacies compared to Tracts with the lowest quartile of poverty (Fig. 2A). Additionally, tracts in the 4th quartile (highest %) of proportion Black/AA residents had a 70.7% shorter average PWSMD to pharmacies than tracts in the 1st (lowest % Black/AA) quartile (Fig. 2C). This trend is similarly mirrored in tracts with increasing Hispanic/Latino populations (Fig. 2D). Conversely, Tracts with residents who had greater educational attainment had larger PWSMD to pharmacies. However, compared to tracts in the first quartile of the proportion of residents who had an Associate’s degree or higher, tracts in the 2nd, 3rd and 4th quartiles (where 4th quartile = higher % of higher educational attainment) of educational attainment all had significantly larger PWSMD, but a trend of greater PWSMD across quartiles was not evident (Fig. 2B). When adjusting for population density, we observe that the aforementioned trends in PWMSD hold (i.e. as disadvantage increases, PWMSD decreases, Supplemental Fig. 1, Supplemental Table 1).

**Table 1:** Descriptive Statistics of Quartile Breakdowns. For each characteristic (% poverty, % higher education, % Black/AA, % Hispanic/Latino) across 4844 tracts in NYS per the ACS 2014–2018 data, we split tracts by quartile and provide the number of census tracts included (“Count”) and the mean, standard deviation, and median population weighted mean shortest distance of these census tracts from pharmacies in kilometers (“Mean (km)”, “Standard Deviation (km)”, and “Median (km)”) by quartile

Category	Count	Mean (km)	Standard Deviation (km)	Median (km)
% Poverty				
1st Quartile (lowest % poverty)	1211	2.17	2.74	1.42
2nd Quartile	1211	2.70	3.91	0.99
3rd Quartile	1211	2.57	4.17	0.72
4th Quartile (highest % poverty)	1211	0.84	1.59	0.49
% Higher Education				
1st Quartile (lowest % higher education)	1211	1.82	3.50	0.60
2nd Quartile	1211	2.68	4.34	0.86
3rd Quartile	1211	2.25	3.22	1.04
4th Quartile (highest % higher education)	1211	1.51	1.63	0.95
% Black/African American (AA)				
1st Quartile (lowest % Black/AA)	1211	3.94	4.89	1.67
2nd Quartile	1211	2.33	3.47	1.10
3rd Quartile	1211	1.29	1.43	0.82
4th Quartile (highest % Black/AA)	1211	0.71	0.91	0.53
% Hispanic/Latino				
1st Quartile (lowest % Hispanic)	1211	4.61	5.14	2.52
2nd Quartile	1211	1.83	2.47	0.98
3rd Quartile	1211	1.20	1.63	0.69
4th Quartile (highest % Hispanic)	1211	0.63	0.63	0.46

**Table 2:** Analysis Results. For each characteristic (% poverty, % higher education, % Black/AA, % Hispanic/Latino) across 4844 tracts in NYS per the ACS 2014–2018 data, we split tracts by quartile provide the calculated % difference in distance to pharmacy from our generalized linear model results (“Estimate”), the 95% CI for that estimate, and the associated *p* value

Term	Estimate (95% CI)	*P* value
**Poverty**		
Intercept/1st Quartile (lowest % poverty)	Referent	
2nd Quartile	-10.0% (-17.1, -2.4)	0.0114
3rd Quartile	-25.7% (-31.6, -19.4)	< 0.001
4th Quartile (highest % poverty)	-61.3% (-64.4, 58.0)	< 0.001
**Higher Education**		
Intercept/1st Quartile (lowest % higher education)	Referent	
2nd Quartile	46.8% (34.7, 59.9)	< 0.001
3rd Quartile	53.2% (40.5, 66.9)	< 0.001
4th Quartile (highest % higher education)	22.7% (12.6, 33.8)	< 0.001
**Black/African American (AA)**		
Intercept/1st Quartile (lowest % Black/AA)	Referent	
2nd Quartile	-37.2% (-42.0, -32.1)	< 0.001
3rd Quartile	-54.4% (-57.8, -50.6)	< 0.001
4th Quartile (highest % Black/AA)	-70.7% (-72.9, -68.3)	< 0.001
**Hispanic**		
Intercept/1st Quartile (lowest % Hispanic)	Referent	
2nd Quartile	-58.1% (-61.0, -58.1)	< 0.001
3rd Quartile	-70.2% (-72.2, -67.9)	< 0.001
4th Quartile (highest % Hispanic)	-80.7% (-82.1, -79.3)	< 0.001

**Fig. 3:**
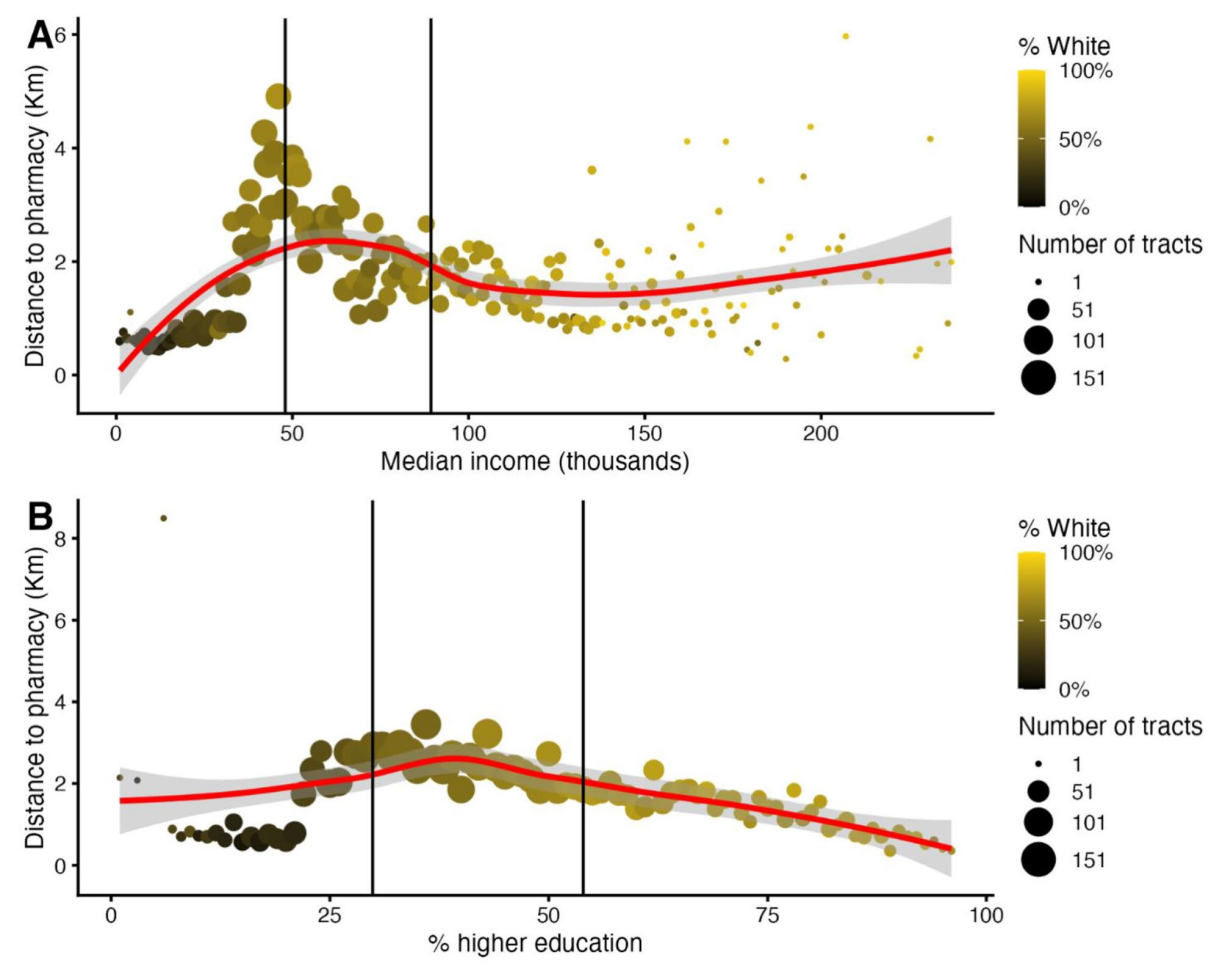
Visual trends in distance to pharmacy. For median income (Panel **A**) and % higher education (Panel **B**) we bin census tracts per the aforementioned characteristics (bins of income increments of $1000 in panel A and bins of % higher education increments of 1% in panel B) and plot the average distance to pharmacies for those tracts as a dot. The size of the dot indicates how many tracts were in that bin. Additionally we color code each dot according to the proportion of white residents in the tracts for that bin. Lastly we indicate the 25th and 75th percentile of the X axis variable (median income or % higher education) with vertical bars. A LOESS curve (in red) with 95% CI (in gray) is fit to this data

We further visualized the association between tract characteristics and distance to pharmacies by binning tracts by median income (Fig. 3A; binned tracts in increments of $1000 of median income) and % higher education attainment (Fig. 3B; binned tracts in increments of 1% of % higher education attainment). We then plot points representing the average distance to a pharmacy for tracts in each of the bins (of the characteristic on the x-axis). The points are sized and colored according to the # of tracts in the bin and % white population in the bin represented by the point, respectively. Lastly a locally weighted scatter-plot smoother curve is fit to the data to help visualize trends. Figure 3A shows that as median income decreases below the 25th percentile of income, the mean distance to pharmacies decreases. From the 25th– 75th percentiles of income, distance to pharmacies also decreases, but plateaus beyond the 75th percentile. When we stratify this plot by city vs. rural tracts (i.e. top 25th percentile of population density tracts vs. less than the top 25th percentile), we see that the trend is largely contributed to by the rural areas (Supplemental Fig. 2A, B). Furthermore, we plot % white of tracts in each bin and see that the tracts that have lower income also have lower % white individuals. The distance to pharmacies increases as income increases (with peak at ∼$50,000 of median income). Figure 3B shows that as higher education attainment decreases below the 25th percentile, the distance to pharmacies also decreases. However, tracts in the higher end of % educational attainment also had a shorter distance to pharmacies compared to tracts at the 25th– 75th percentile of % educational attainment range (dots between vertical lines). This trend is also reflected when tracts are stratified by rural vs. city tracts (Supplemental Fig. 2A, B). Once again, tracts that had lower education attainment were predominantly non-white.

## Discussion

Overall, our analyses show that in New York state there is a trend of higher spatial access to pharmacies across tract-level quartiles of increasing poverty rate and increasing proportions of residents who were Black/AA or Hispanic/Latino. Furthermore, we find that tracts in the 2nd to 4th quartile of proportion of residents with higher educational attainment, had lower spatial access to pharmacies than tracts in the first quartile. These findings are contrary to the hypothesis that pharmacy access might serve as a neighborhood-level mechanism of FCT, that is, low-income communities and Black and Hispanic communities would have decreased access to pharmacies.

The prior literature on spatial access to pharmacies has typically focused on single cities and has found inconsistent results. Analyzing the locations of chain pharmacies in relation to geometric centers of neighborhood blocks, Guadamuz et al. showed that there is an increase in pharmacy deserts (which they principally define as areas with increased neighborhood centroid distance to pharmacies) in Black & Latino neighborhoods of New York City, Houston, Los Angeles, and Chicago [16]. They also found similar trends in pharmacy desert prevalence in a separate analysis where they defined a desert as a neighborhood ≥ 1 mi from a pharmacy (or ≥ 0.5 mi if the neighborhood had ≥ 100 households with no vehicle access). In contrast, Ikram et al. showed that within Baton Rouge, Louisiana, pharmacies are located closer to African American neighborhoods [17]. Their analysis used a 2-step floating catchment area method that used a supply-demand metric to quantify accessibility (where lower demand is indicated by higher driving time from a neighborhood centroid to a pharmacy). Many other studies (as systematically reviewed by Jagadeesan et al.) have also explored the usage of Geographic Information Systems (GIS) in determining spatial relationships between population density and specific pharmaceutical services (such as vaccinations, specific medicines, syringes, blood pressure monitoring, etc.) [18]. Their review of twenty studies from 2000 to 2018 included studies that used aggregated rural and urban data (6/20) at the national level or at the state level (8/20). Additionally, they noted that 14/20 studies used simple Euclidean distances from neighborhood centroids.

Our study addresses shortcomings of the previously analyses of pharmacy data by including a large variety of urban and non-urban regions across an entire state, accounting for access to pharmacies across state borders and analyzing chain and non-chain pharmacies. The studies by Guadamuz and Ikram both focused on cities as the unit of analysis and analyzed only chain pharmacies. In addition, the work presented here uses a population weighted measure of the shortest street network distance to pharmacies, whereas many prior analyses used a simple Euclidean distance metric without population weights. The population weighted measures used here is thought to better reflect the population experience for pharmacy access. Furthermore, these studies used binary measures of demographic characteristics, such as Guademuz’s classification of tracts as being “Black neighborhoods”, whereas the use of quartile cut-points allowed for analyses of trends in spatial access.

Our study is consistent with the prior literature in observing that rural populations typically have longer distances to suburban pharmacies and additionally show that city centers contain the majority of pharmacies in analyzed areas. This urban/rural disparity is thought to reflect less demand in rural areas and perceived social isolation among pharmacy employees in less populous locations [26]. Furthermore, economic pressures from large chain pharmacies and reduced insurance reimbursements for prescriptions have made it difficult for rural pharmacies to stay open [27]. Prior studies have found that, while all racial groups in America have become increasingly suburbanized, white populations are more likely to live in suburbs [28, 29]. Exclusionary zoning in suburbs reduces land-use mix and so pharmacies are located in retail and commercial areas removed from the residential neighborhoods. However, higher private automobile ownership rates in the suburbs means that pharmacies in these areas can “afford” to be further away from the individuals they serve. In contrast urban centers, often with higher poverty rates and larger communities of color, tend to have mixed land-use zoning which places retail in closer proximity to residences. Furthermore, lower access to private automobiles and a greater dependence on walking and public transit incentivizes a higher density of retail, including pharmacies, in urban areas.

### Limitations

A limitation of this study is that it focuses only on spatial access to pharmacies and does not address the experience of using pharmacies in different areas of NYS and the extent to which pharmacies differ in the health services they provide. It is possible that pharmacies in lower income areas are less well stocked, less well staffed and may not provide as extensive health services as those in higher income or suburban areas [30]. The relatively higher spatial access to pharmacies in lower income areas may be off set if the quality of the pharmacies in these areas is lower. Additionally, inequities in pharmacist practice have been noted to be prevalent in minority predominant settings [31]. Given the scale of the analysis and the thousands of pharmacies in New York State, it would be difficult to establish a common, standardized metric for extent of services. This study also does not consider usage of mail-order prescriptions which may off-set lower spatial access to pharmacies. Use of mail-order prescriptions has been found to be associated with non-Hispanic White race/ethnicity, higher education attainment and higher income, suggesting use of mail-order is higher in areas found to have lower spatial access in our study [32]. However, pharmacies can provide access to other health promoting services that cannot be replicated via mail-order. Lastly, we acknowledge that these analyses are ecologic and do not address the Multiple Areal Unit Problem (MAUP) and thus may not generalize to the level of individuals or other spatial units that could have been analyzed such as ZIP Codes or counties [33]. 

### Significance

The primary significance of this study is that it includes analyses that cover a large, diverse geographic area, a novel measure of spatial access and allowing for the access measure to cross state lines. Past studies of spatial access to (diss)amenities such as fast food, grocery stores, park space, and medical establishments have often estimated counts or densities of establishments within administrative boundaries such as tracts, ZIP Codes or counties. These metrics suffer from boundary issues, uncertain geographic context problems, the modifiable areal unit problem and do not account for differences in street connectivity [34]. Through the use of a population weighted distance measures our spatial accessibility metric seeks to describe the shortest street network distance experienced by the population of a tract, allowing for pharmacies outside of the tract to contribute to spatial access and accounting for differences in street connectivity.

## Conclusion

We find that tracts with higher poverty rates and higher proportions of Black or African American residents and higher proportions of Hispanic residents have higher spatial access to pharmacies in NYS. This work suggests that lower income communities and communities of color have higher access to the health benefits of pharmacies, as well as to disamenities, contrary to what is predicted by fundamental cause theory.

### Electronic supplementary material

Below is the link to the electronic supplementary material.

Supplementary Material 1

## Data Availability

The data used in this study is available here: https://drive.google.com/file/d/1YbQgv2MRCxG9QGRiNFQjIuXHa0D3JbIq/view?usp=share_link Code is available at the following link: https://drive.google.com/file/d/15brx0dDQlWgWvSHOdA9kWTfmW_n45a7D/view?usp=sharing.

## Abbreviations

NYS: New York State
PWMSD: Population weighted mean shortest (road) network distance
AA: African American
SD: Standard Deviation
CI: Confidence Interval
FCT: Fundamental Cause Theory
SES: Socioeconomic status
GLM: Generalized Linear Model
