# Supplementary material for: Disadvantaged groups have greater spatial access to pharmacies in New York state
Source: BMC Health Serv Res. 2024 Apr 15;24:471. doi: 10.1186/s12913-024-10901-8 (PMC11017547; doi:10.1186/s12913-024-10901-8)

**Supplemental Materials**

**Section 1: Pharmacy Access Measurement**

To examine the association between neighborhood income, poverty, and racial disparities in access to pharmacies, a measure of local access was needed. Our measure consisted of computing the shortest street network travel-distance between each census block centroid in NYS, and the nearest pharmacy using ArcGIS Pro v2.x. Then using these distances for each Census block within each Census Tract, the population weighted mean shortest distance across Blocks was calculated for each Tract, using the populations of the Census blocks to weight each Census block’s shortest distance measure. The resulting dataset is made public for use in the link provided in our manuscript. In comparison with other methods of calculating geographical accessibility, this measure is thought to better express the population experience of distance to pharmacies because it accounts for the distribution of the population inside Census tracts. Also as compared to other “container based methods” (e.g. count or density of pharmacies in a tract) this method includes distances to pharmacies outside of Tracts and in neighboring states. For residents of some Census blocks in a given Tract, the closest pharmacy might be in a neighboring Tract or in a neighboring state and our approach accounts for these possibilities. We chose Tracts as the unit of analysis for several reasons: 1) Tract boundaries are more stable year to year that ZIP Code boundaries; 2) ZIP Codes often have irregular boundaries; 3) ZIP Codes vary a lot in their population size across NY State; and 3) a lot of geospatial and health data in New York State is provided at the Tract-level and so this is a unit that policy makers are accustomed to seeing and working with. However, this method can also be applied to ZIP Code Tabulation Areas (ZCTAs) using Census blocks nested in ZCTA. This approach can be used for measuring spatial access to other neighborhood amenities such as supermarkets or medical facilities or to disamenities such as abandoned buildings, vacant lots or polluting facilities.

In terms of analysis using GLM, we provide the following code for use at <https://drive.google.com/file/d/15brx0dDQlWgWvSHOdA9kWTfmW_n45a7D/view?usp=sharing> .

**Section 2: Analyses adjusting for population density**

To formally analyze the effect of population density on our analysis, we re-ran the analyses controlling for population density as a continuous variable. We present a figure (**Supplemental Figure 1)** and a table (**Supplemental Table 1**) with the results from this analysis.

**Supplemental Figure 1: Population Density adjustment analysis plot:** For each of the characteristics examined (A = % poverty, B = % higher education, C = % Black/African American, D = % Hispanic/Latino) from 5-year estimate ACS data across 4844 census tracts in New York State, we plot the % differences in distance to pharmacies from our generalized linear model analysis. Since 1st quartile was treated as the reference level in our analyses, this level was excluded from this graph. Null effect is indicated with the vertical dashed line and the coefficients with associated 95% CIs are plotted (dot = coefficient value, lines = 95% CI).


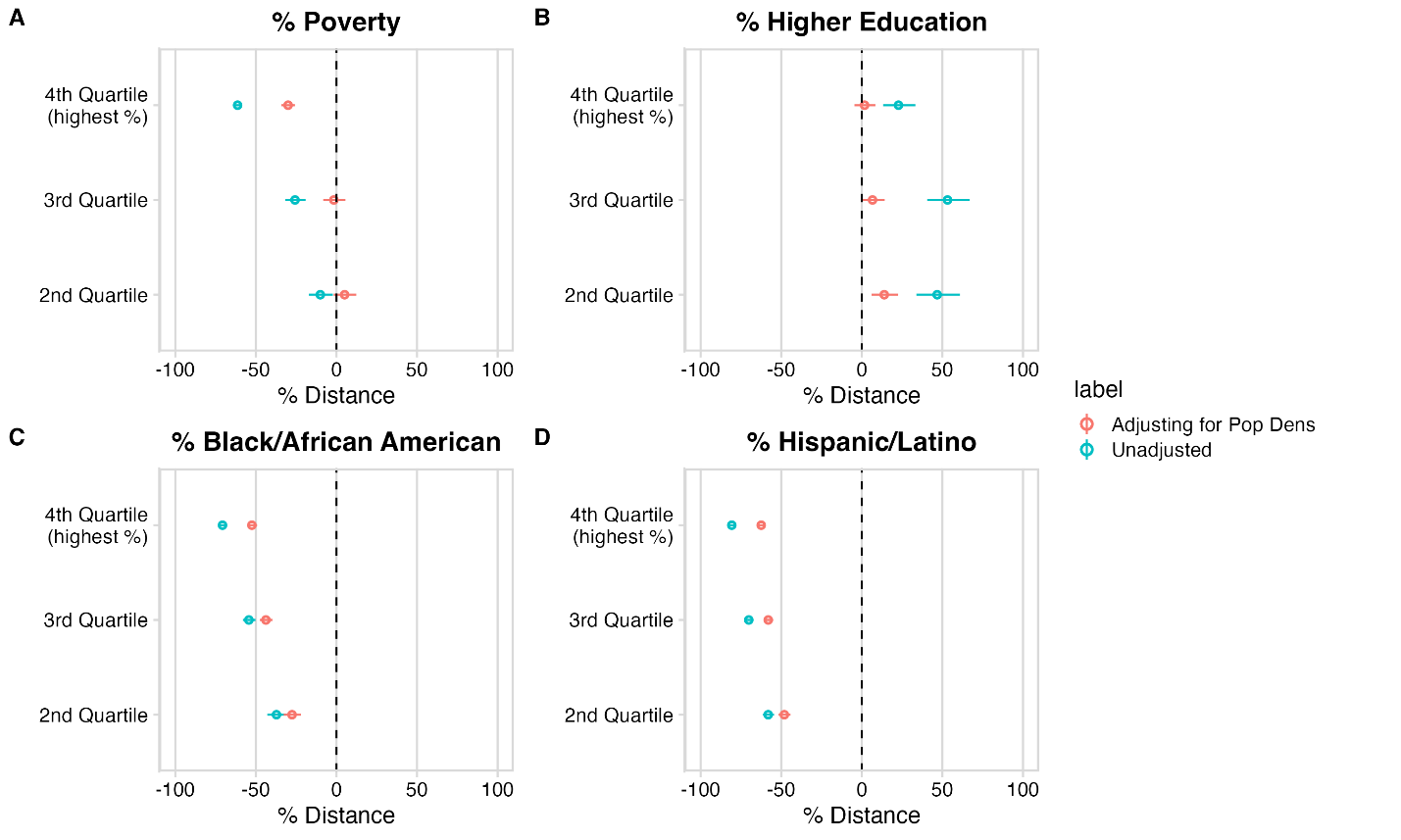


**Supplemental Table 1: Population Density analysis table:** For each characteristic (% poverty, % higher education, % Black/AA, % Hispanic/Latino) across 4844 tracts in NYS per the ACS 2014-2018 data, we split tracts by quartile provide the calculated % difference in distance to pharmacy from our generalized linear model results (“Estimate”) and the 95% CI for that estimate.

| Coefficient name | Mean (%) | CI upper (97.5%) | CI lower (2.5%) | Adjusted mean (%) | Adjusted CI upper (97.5%) | Adjusted CI lower (2.5%) |
| --- | --- | --- | --- | --- | --- | --- |
| Quartile Poverty 2nd | -10.03 | -17.16 | -2.29 | 5.06 | -1.72 | 12.32 |
| Quartile Poverty 3rd | -25.71 | -31.77 | -19.12 | -1.60 | -8.27 | 5.56 |
| Quartile Poverty 4th | -61.35 | -63.82 | -58.71 | -30.02 | -34.06 | -25.73 |
| Quartile Higher Ed 2nd | 46.76 | 33.85 | 60.91 | 13.91 | 5.94 | 22.48 |
| Quartile Higher Ed 3rd | 53.17 | 40.57 | 66.89 | 6.62 | -0.44 | 14.19 |
| Quartile Higher Ed 4th | 22.74 | 13.13 | 33.17 | 1.70 | -4.61 | 8.43 |
| Quartile Black/AA 2nd | -37.23 | -42.90 | -31.01 | -27.56 | -32.75 | -21.97 |
| Quartile Black/AA 3rd | -54.36 | -58.11 | -50.28 | -43.78 | -47.46 | -39.85 |
| Quartile Black/AA 4th | -70.71 | -72.93 | -68.30 | -52.49 | -55.51 | -49.26 |
| Quartile Hispanic 2nd | -58.11 | -61.46 | -54.48 | -48.10 | -51.68 | -44.26 |
| Quartile Hispanic 3rd | -70.15 | -72.43 | -67.69 | -58.09 | -60.91 | -55.07 |
| Quartile Hispanic 4th | -80.71 | -82.06 | -79.25 | -62.45 | -64.96 | -59.75 |

**Supplemental Figure 2: Visual trends in distance to pharmacy.** For median income (Panel A, B) and % higher education (Panel C, D) we bin census tracts per the aforementioned characteristics (bins of income increments of $1000 in panel A/B and bins of % higher education increments of 1% in panel C/D) and plot the average distance to pharmacies for those tracts as a dot. The size of the dot (“ct” on legend) indicates how many tracts were in that bin. Additionally we color code each dot according to the proportion of white residents in the tracts for that bin (“m” on legend). We indicate the 25^th^ and 75^th^ percentile of the X axis variable (median income or % higher education) with vertical bars. A LOESS curve (in red) with 95% CI (in gray) is fit to this data. Type indicates whether or not the graph is only looking at tracts that are City tracts (top 25^th^ percentile population density) or Rural (bottom 75^th^ percentile population density).


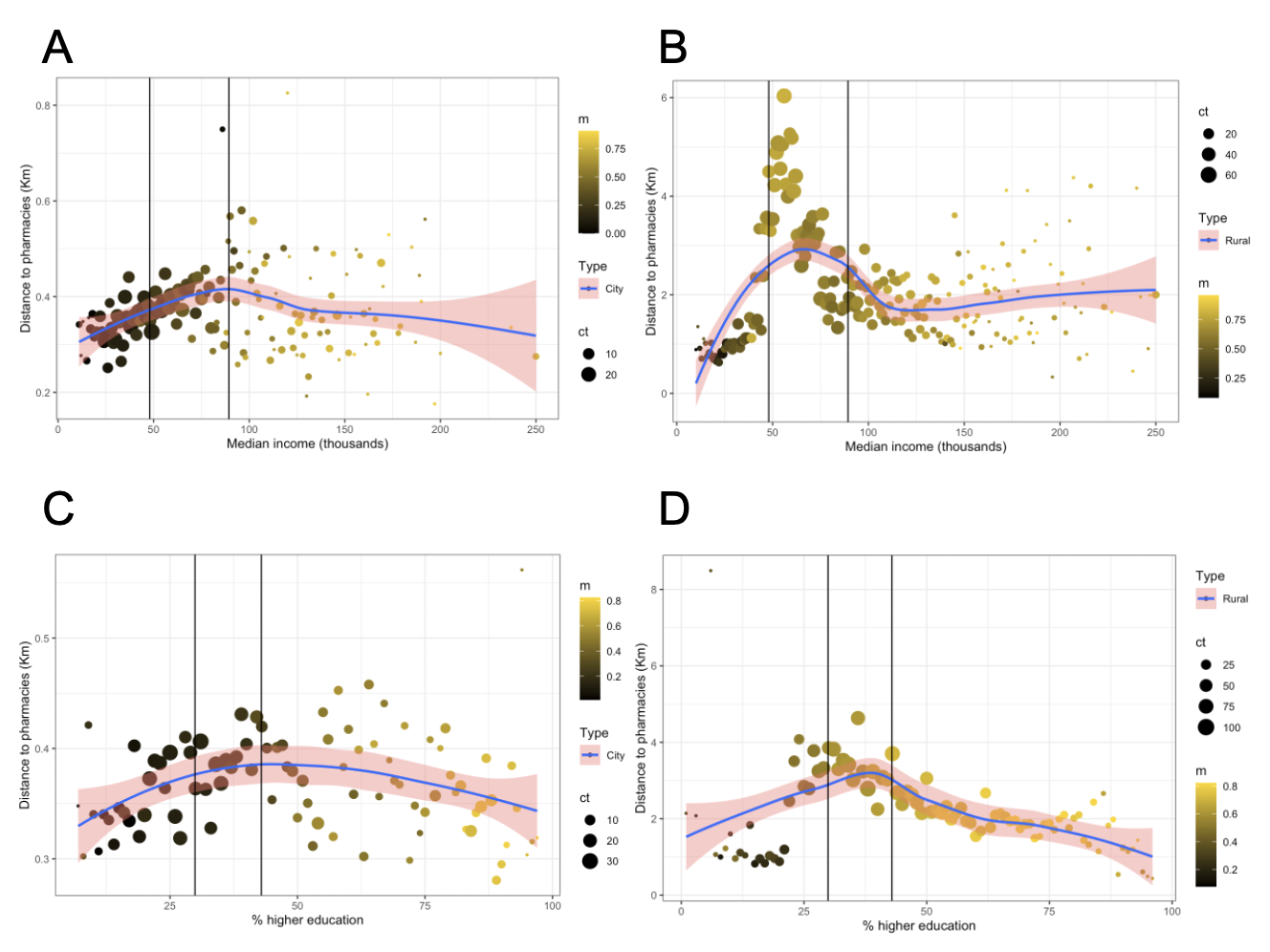


**Supplemental Section 3: Assessment of spatial auto-correlation and spatially lagged models:**

Given the structure of the data set we expected a priori that spatial autocorrelation would be present in the data, an expectation that is supported by the initial Moran’s I tests. Spatial autocorrelation often arises when there are spatially distributed confounding variables. As noted in the main paper the goal of these analyses was to document disparities in access to pharmacies, but not explain the causes of these disparities. However, here we present results from spatially lagged models in which for each tract we adjust for the measured distance to pharmacies of the neighboring tracts. In the spatially lagged models the Moran’s I is non-significant, however we do not interpret these results to suggest that pharmacy access in the adjoining tracts is a spatially distributed causal confounder. Rather, as described in the Discussion of the main paper, we believe that zoning, which impacts land use mix, is the most likely spatially distributed confounder that explains the observed disparities and that zoning is associated with the values of the sociodemographic variables in each tract and with distance measures in the neighboring tracts.

Below we present results from spatially lagged models. For each model (poverty, higher education (ed), Black/AA, Hispanic), we calculate a Moran’s I and calculate skewness for each spatially lagged model. We also provide the unadjusted (original) and adjusted (spatially lagged model) coefficients (“coef.b”) with confidence intervals (lower bound: “2.5%”, upper bound:“97.5%”) and converted percentages (“pct”) with confidence intervals (lower bound: “pct25”, upper bound: “pct975”) for each individual tract. For all of the below models, the Moran’s I after adjusting (spatially lagged model) is no longer significant.

**Poverty model:**

**Moran I test under randomization**

Moran I statistic standard deviate = -9.928, p-value = 1

alternative hypothesis: greater

sample estimates:

Moran I statistic Expectation Variance

-8.505777e-02 -2.065262e-04 7.304494e-05

**D'Agostino skewness test**

skew = -0.52767, z = -14.13123, p-value < 2.2e-16

alternative hypothesis: data have a skewness

Unadjusted:


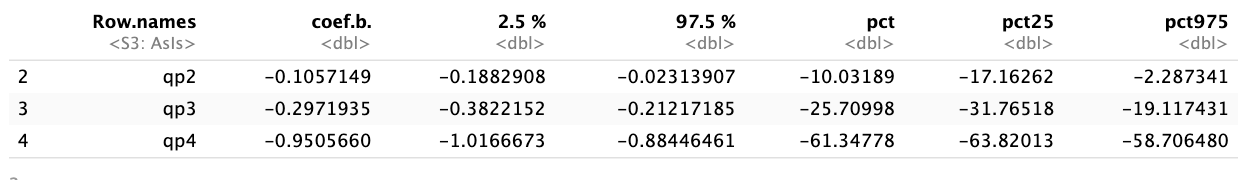


Adjusted:


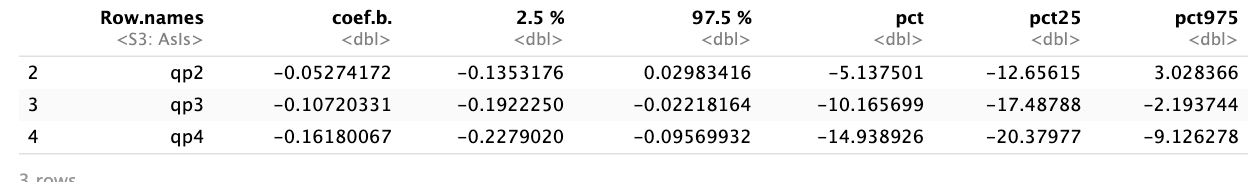


**Higher Ed model:**

**Moran I test under randomization**

Moran I statistic standard deviate = -10.508, p-value = 1

alternative hypothesis: greater

sample estimates:

Moran I statistic Expectation Variance

-9.001828e-02 -2.065262e-04 7.304421e-05

**D'Agostino skewness test**

skew = -0.56791, z = -15.08264, p-value < 2.2e-16

alternative hypothesis: data have a skewness

Unadjusted:


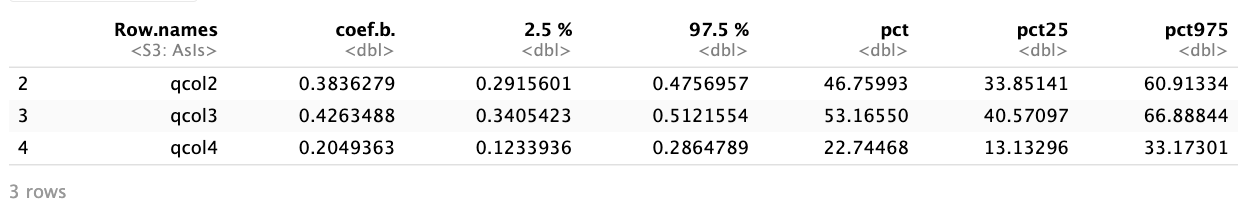


Adjusted:


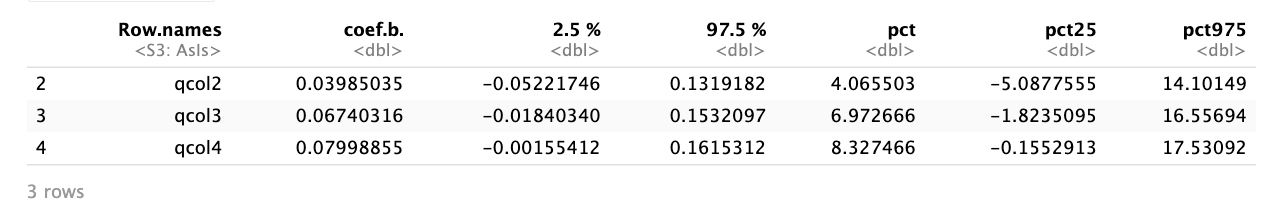


**Black/AA model:**

**Moran I test under randomisation**

Moran I statistic standard deviate = -10.356, p-value = 1

alternative hypothesis: greater

sample estimates:

Moran I statistic Expectation Variance

-8.871181e-02 -2.065262e-04 7.304332e-05

**D'Agostino skewness test**

skew = -0.59564, z = -15.72593, p-value < 2.2e-16

alternative hypothesis: data have a skewness

Unadjusted:


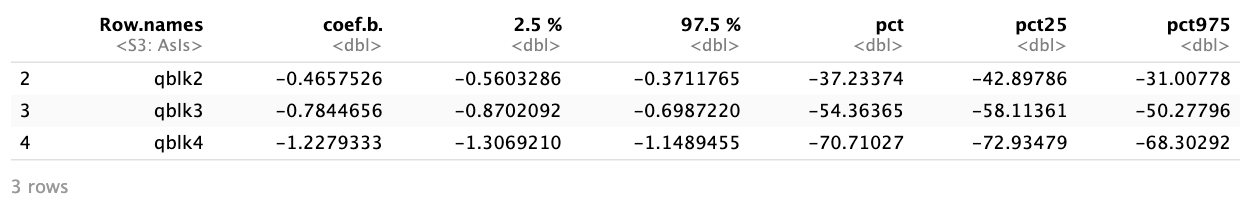


Adjusted:


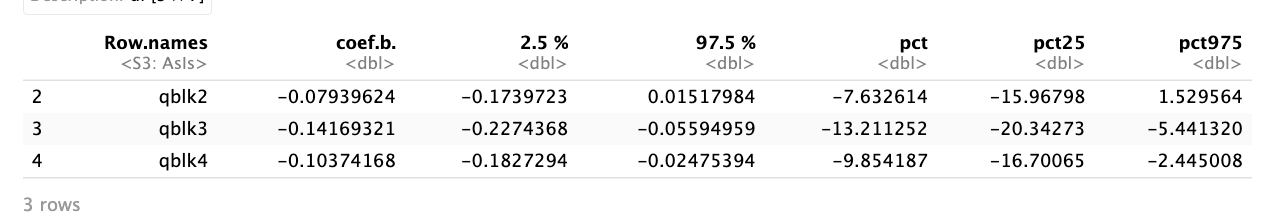


**Hispanic model:**

**Moran I test under randomisation**

Moran I statistic standard deviate = -9.7007, p-value = 1

alternative hypothesis: greater

sample estimates:

Moran I statistic Expectation Variance

-8.311403e-02 -2.065262e-04 7.304389e-05

**D'Agostino skewness test**

skew = -0.58964, z = -15.58750, p-value < 2.2e-16

alternative hypothesis: data have a skewness

Unadjusted:


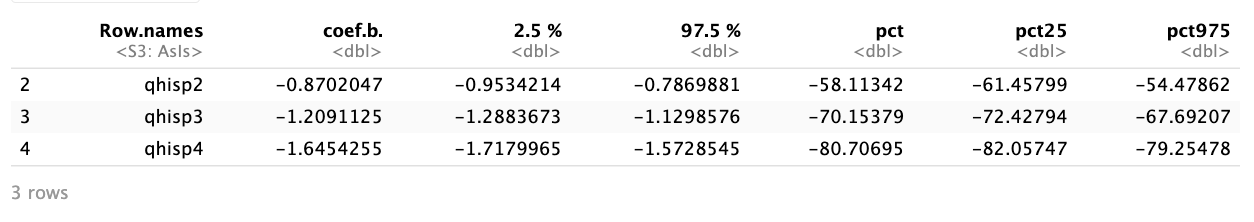


Adjusted:


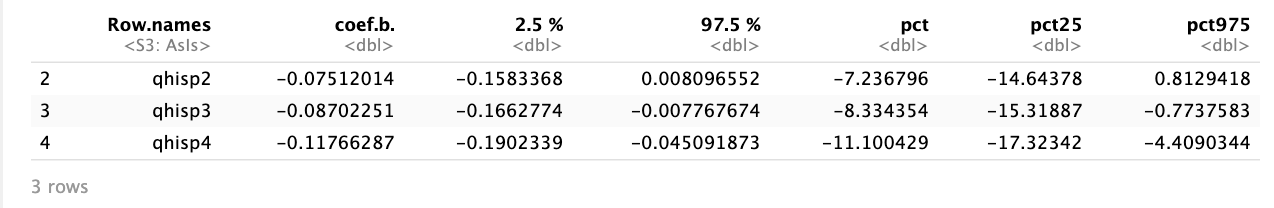

Supplement: Supplementary file 1 — Supplementary Material 1 [file 12913_2024_10901_MOESM1_ESM.docx]
